# Molecular Mechanisms of Root Development in Rice

**DOI:** 10.1186/s12284-018-0262-x

**Published:** 2019-01-10

**Authors:** Funing Meng, Dan Xiang, Jianshu Zhu, Yong Li, Chuanzao Mao

**Affiliations:** State Key Laboratory of Plant Physiology and Biochemistry, College of Life Sciences, Zhejiang University, Hangzhou, 310058 China

**Keywords:** Rice, Root system architecture, Molecular mechanism, Genetic control

## Abstract

Roots are fundamentally important for growth and development, anchoring the plant to its growth substrate, facilitating water and nutrient uptake from the soil, and sensing and responding to environmental signals such as biotic and abiotic stresses. Understanding the molecular mechanisms controlling root architecture is essential for improving nutrient uptake efficiency and crop yields. In this review, we describe the progress being made in the identification of genes and regulatory pathways involved in the development of root systems in rice (*Oryza sativa* L.), including crown roots, lateral roots, root hairs, and root length. Genes involved in the adaptation of roots to the environmental nutrient status are reviewed, and strategies for further study and agricultural applications are discussed. The growth and development of rice roots are controlled by both genetic factors and environmental cues. Plant hormones, especially auxin and cytokinin, play important roles in root growth and development. Understanding the molecular mechanisms regulating root architecture and response to environmental signals can contribute to the genetic improvement of crop root systems, enhancing their adaptation to stressful environmental conditions.

**Electronic supplementary material:**

The online version of this article (10.1186/s12284-018-0262-x) contains supplementary material, which is available to authorized users.

## Background

A major challenge for plants is the complexity of the environment in which they must survive. Root systems are vital for addressing this complexity. Roots not only provide structural support to the aerial organs of the plant, and enable the acquisition of water and nutrients that are required for plant growth, but also can monitor environmental conditions in the soil, such as the water content, nutrient levels, and the presence of toxic elements. To ensure an optimal response to changing environmental situations, root systems are continuously reshaped by the initiation and elongation of new roots throughout the growth period, facilitating the plant’s adaptation to biotic and abiotic stresses (Fukai and Cooper 1995; Gowda et al. 2011). Understanding the mechanisms that control root patterning and identifying the genes responsible for post-embryonic root initiation could therefore enable breeders to improve crop tolerance to abiotic stresses (Coudert et al. 2010).

There are two main types of root systems in plants, defined by their developmental origin and branching patterns: taproot systems and fibrous root systems. The taproot system occurs in dicot plants such as *Arabidopsis thaliana*, tomato (*Solanum lycopersicum*), and pea (*Pisum sativa*), while fibrous root systems occur in monocots such as rice (*Oryza sativa*), wheat (*Triticum aestivum*), and maize (*Zea mays*) (Atkinson et al. 2014). In taproot systems, the primary root is the main root, and branching consists of secondary, smaller lateral roots (LRs) and root hairs. A fibrous root system, by contrast, consists of a dense mass of adventitious roots (also called crown roots in cereals) that arise from the stem, which are distinct from the primary root, LRs, and root hairs (Coudert et al. 2010). Because the primary root (embryonic root) dies as the monocots age, the adventitious roots are the main root tissues in the fibrous root system of monocot plants.

Rice is a monocot model plant that provides a good experimental system for addressing the molecular mechanisms of constitutive and adaptive root branching and development in fibrous root systems (Gojon et al. 2009). The genetic regulatory network of root development in *Arabidopsis* is not suitable for understanding this process in rice which, unlike *Arabidopsis*, forms many adventitious roots. Deciphering the molecular regulatory network of root development in rice is therefore vital for enhancing our understanding of the genetic regulation of root development in the monocots more generally. Furthermore, a thorough knowledge of the key genes involved in rice root development will enable breeders to improve the root system and generate high-yield and nutrient-efficient cultivars using genetic manipulation or marker-assisted selection.

With the rapid development of functional genomics, significant progress has been made in elucidating the genetic control of root development in rice using mutant and quantitative trait loci (QTL) analyses. Here, we review the current progress in identifying genes regulating the development of the rice root system, including the primary root, crown root, lateral root, and root hairs. This research will improve our understanding of root system architecture (RSA) development and ultimately enable breeders to select for ideal root architectures promoting higher-yielding crops.

## Main Text

### Genes Regulating Root Length

Root length is an important component of root architecture that is essential to survival in complex soil conditions. A number of QTLs have been found to play major roles in regulating root growth and development in rice (Zhang et al. 2001; Toorchi et al. 2002, 2007; Obara et al. 2010; Wang et al. 2013; Li et al. 2015a), many of which were summarized in a previous review (Coudert et al. 2010); however, most of these QTLs have not been cloned. Recently, a QTL controlling root thickness and length, *qRT9*, was found to encode a basic helix-loop-helix (bHLH) transcription factor, OsbHLH120. *OsbHLH120* expression was strongly induced by polyethylene glycol, salt, and the drought-response hormone abscisic acid (ABA), suggesting an association with drought avoidance (Li et al. 2015a).

In addition to these QTLs, several genes regulating root length have been identified in mutant analyses. The rice UDP-N-acetylglucosamine biosynthesis-related gene *OsGNA1* controls root elongation, as the loss-of-function mutant *Osgna1* exhibited a temperature-sensitive defect in root elongation, with disrupted microtubules and cell shrinkage in the root elongation zone (Jiang et al. 2005). A putative mannosyl-oligosaccharide glucosidase (OsMOGS), required for N-glycan maturation, was also found to regulate root elongation by affecting cell division and elongation (Wang et al. 2014a). Additionally, two sugar metabolism-related genes were also found to be involved in root development. *OsDGL1* encodes a DOLICHYL DIPHOSPHOOLIGOSACCHARIDE-PROTEIN GLYCOSYLTRANSFERASE 48 kDa subunit precursor, which has conserved functions with the oligosaccharyltransferase complex found in all eukaryotes; mutations in this gene cause a defect in N-glycosylation in the root, resulting in shorter root cells, smaller root meristems, and root cell death (Qin et al. 2013). *OsCYT-INV1,* which encodes an alkaline/neutral invertase, also plays an important role in root elongation, as the *Oscyt-inv1* mutant produces short roots. *Oscyt-inv1* was found to accumulate sucrose and had reduced levels of hexose; however, its short-root phenotype could be rescued by exogenously supplying glucose (Jia et al. 2008). These studies suggest that genes involved in sugar metabolism and the sugar-based modification of proteins play important roles in root elongation.

Recent studies have demonstrated that root elongation is also associated with cell wall biosynthesis. *OsGLU3* encodes a putative membrane-bound endo-1,4-β-glucanase, which is necessary for root elongation in rice. The *Osglu3* mutant produced short roots with lower cellulose contents in its root cell walls, while the exogenous application of glucose suppressed these phenotypic defects (Zhang et al. 2012)*.* Moreover, silencing the rice α-expansin gene *OsEXPANSION 8* (*OsEXPA8*), which encodes a cell wall-localized protein expressed predominantly in the root and shoot, resulted in a shorter primary root, fewer LRs, and short root hairs, supporting the hypothesis that expansins are involved in root growth by mediating cell wall loosening (Wang et al. 2014b).

Furthermore, genes involved in a range of physiological pathways have also been shown to regulate root length in rice. A T-DNA insertion mutant of a rice glutamic acid receptor-like gene, *OsGLR3.1*, was found to produce a short root, and further analysis revealed that this gene is essential for the maintenance of cell division and survival in the root apical meristem (RAM) in early seedlings (Li et al. 2006). *OsGatB* encodes a subunit of tRNA-dependent amidotransferase, an essential enzyme involved in Gln-tRNA^Gln^ biosynthesis in mitochondria, and may promote primary root growth by maintaining mitochondrial structure and function to facilitate cell division and elongation in the root tip (Qin et al. 2016). OsASL1, an argininosuccinate lyase that catalyses the final step of arginine biosynthesis, is also required for primary root elongation in rice, suggesting that a specific concentration of arginine is required for normal root growth in rice (Xia et al. 2014). OsELICITOR 5 (OsEL5), a membrane-anchored RING-H2-type ubiquitin E3 ligase, maintains cell viability after the initiation of root primordia (Koiwai et al. 2007). In addition, OsSPR1, a mitochondrial protein with an Armadillo-like repeat domain, is involved in post-embryonic root elongation and ion homeostasis (Jia et al. 2011), while *OsMYB1*, encoding an R2R3-type transcription factor, regulates primary root elongation in a phosphate-dependent manner (Gu et al. 2017).

Certain phytohormone-related genes can also regulate rice primary root growth. The auxin transporter mutant *Osaux1* had a longer primary root and shorter root hairs than the wild type (WT) when grown in hydroponic culture (Yu et al. 2015), while knock-out lines of *OsAUXIN RESPONSE FACTOR 12* (*OsARF12*) had shorter primary roots (Qi et al. 2012). The microRNA *miR393* influences auxin signalling to mediate primary root and adventitious root development by regulating its target genes, *OsTIR1* and *OsAFB2*, the rice orthologs of the *Arabidopsis* auxin receptors TRANSPORT INHIBITOR RESPONSE 1 (TIR1) and AUXIN SIGNALING F-Box 2 (AFB2), which interact with OsIAA1, an AUXIN/INDOLE ACETIC ACID (AUX/IAA) regulatory protein (Bian et al. 2012). Moreover, silencing the expression of *DNA TOPOISOMERASE 1* (*TOP1*), an essential manipulator of DNA topology during RNA transcription and DNA replication, strongly reduced rice root elongation and gravitropism by mis-regulating auxin signalling and its associated transporters (Shafiq et al. 2017). Ethylene (ET) also appears to be involved in root development in rice. OsEIL1, a transcription factor involved in the ethylene signalling pathway, promotes rice root elongation (Mao et al. 2006). In addition, a recent study showed that the gain-of-function mutant *Osethylene responsive factor 2* (*Oserf2*) formed a shorter root than the WT, while silencing *OsERF2* leads to a long-root phenotype. Further experiments revealed that *OsERF2* is required for the root response to both ABA and ethylene signalling (Xiao et al. 2016). Another gene, *SHB*, which encodes an AP2/ERF transcription factor, affects gibberellic acid biosynthesis, as well as the elongation and proliferation of root meristem cells (Li et al. 2015b). Additionally, recent studies revealed that strigolactones (SL) are required for the induction of root elongation by nitric oxide in response to nitrogen and phosphate deficiencies in rice (Sun et al. 2016). A salicylic acid biosynthesis-related gene, *OsAIM1*, is also required for root growth in rice, through promoting reactive oxygen species (ROS) accumulation (Xu et al. 2017).

**Fig. 1:**
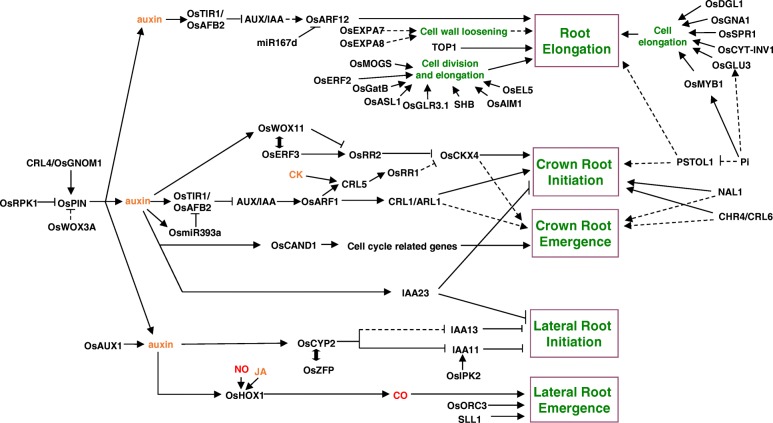
The molecular regulatory mechanisms of root development in rice. Arrows represent positive regulatory actions. Lines ending in a flat head indicate a negative regulatory action. Dashed lines represent interactions that have not been experimentally confirmed. Double-headed arrows indicate that two proteins interact. Text color code: genes or protein, black; hormones, yellow; signals, red; biological processes, green

Although about 20 genes required for root elongation have been identified, most function in different genetic pathways (Fig. 1); therefore, further research is still required to elucidate the molecular regulatory mechanisms of root elongation in rice.

### Molecular Mechanisms of Crown Root (Adventitious Root) Development

Crown roots are one of the most important components of fibrous root systems, but are absent from taproot systems. In rice, the crown root primordium originates from the innermost ground meristem cells adjacent to the peripheral cylinder of vascular bundles in the stem (Itoh et al. 2005).

The first crown root development gene to be identified was *ADVENTITIOUS ROOTLESS 1*/*CROWN ROOTLESS 1* (*ARL1/CRL1*), encoding a LOB-domain transcription factor (Inukai et al. 2005; Liu et al. 2005); the knock-out mutant *arl1*, which carries a 20-bp deletion, produced no adventitious roots or adventitious root primordia throughout its entire lifespan (Liu et al. 2005), while the allelic line *crl1*, containing a single amino acid change, occasionally produced crown roots at later developmental stages (Inukai et al. 2005). The shoots of the *arl1/crl1* mutants are identical to the WT, however, while fewer LRs were observed on the primary root of the *crl1* muant in comparison with the WT, the LR numbers on the primary root of *arl1* were not significantly affected (Inukai et al. 2005; Liu et al. 2005). Furthermore, *ARL1/CRL1* is a direct target of OsARF1, which can be induced by exogenous auxin treatment (Inukai et al. 2005). These results indicate that ARL1/CRL1 is a specific regulator of crown roots, with a central role in crown root initiation. Further research is required to elucidate how ARL1/CRL1 regulates crown root development.

Physiological studies have provided additional evidence of auxin playing an important role in crown root development, and the growth of functional genomics has meant that growing numbers of auxin-related genes have been found to be involved in this developmental process. Plants overexpressing the auxin biosynthesis gene *OsYUCCA1* have a higher auxin content, which leads to more crown roots (Yamamoto et al. 2007). Two crown rootless mutants, *crown rootless 4* (*crl4*) and *Osgnom1*, were found to be allelic lines with mutations in a membrane-associated guanine-nucleotide exchange factor of the ADP-ribosylation factor G protein (GNOM). GNOM1 regulates the traffic of PIN-FORMED 1 (PIN1) auxin efflux carrier proteins, and consequently mediates polar auxin transport, suggesting that appropriate polarized auxin transport mediated by CRL4/OsGNOM1 is required for crown root initiation (Kitomi et al. 2008; Liu et al. 2009). Consistant with this, reducing the expression of *OsPIN1* using RNA interference inhibited adventitious root development, a phenotype that could be rescued by the exogenous application of α-naphthylacetic acid (α-NAA), suggesting a role for OsPIN1 in the regulation of adventitious root development via auxin pathway (Xu et al. 2005). Overexpressing *OsRPK1*, which encodes a leucine-rich repeat receptor-like kinase (LRR-RLK), resulted in undeveloped adventitious roots and LRs, and a reduced RAM caused by lower expression of most *OsPIN* genes, suggesting that OsRPK1 also functions in an auxin-related pathway (Zou et al. 2014). Moreover, the gain-of-function mutant *Osiaa23*, which accumulates an auxin response protein, had defects in the initiation of its crown roots and LRs, and maintenance of the quiescent center (QC) in the root tip (Ni et al. 2011). Recently, *NARROW LEAF 1* (*NAL1*), was also found to mediate rice crown root development, as the loss-of-function mutant *nal1* produced fewer adventitious roots. *NAL1* encodes a putative trypsin-like serine/cysteine protease that affects the expression levels of many genes associated with leaf development and auxin transport; consequently, exogenous auxin treatment rescued the *nal1* phenotype (Cho et al. 2014). Another auxin signalling gene, *OsCAND1*, named after its *Arabidopsis* homolog *CULLIN-ASSOCIATED AND NEDDYLATION-DISSOCIATED 1* (*CAND1*), is also required for the emergence of crown roots (Wang et al. 2011). In addition, *CHROMATIN REMODELING 4* (*CHR4*; Zhao et al. 2012), also named *CROWN ROOTLESS 6* (*CRL6*; Wang et al. 2016), encodes a member of the large chromodomain, helicase/ATPase, and DNA-binding domain protein family, and is known to affect both auxin signalling and crown root development in rice. *OsCHR4*/*CRL6* is most highly expressed in the basal region of the stem where crown roots are initiated. The defective crown root formation in *crl6* can be rescued by auxin treatment, and furthermore, the expression of the *OsIAA* genes was down-regulated in *crl6*, providing evidence that *OsCHR4*/*CRL6* plays a role in crown root development through the auxin-signalling pathway (Wang et al. 2016).

Cytokinins (CK) are also essential for crown root development in rice. The WUSCHEL-related homeobox gene *OsWOX11,* expressed in emerging crown roots and cell division regions of the root meristem, is an important player in CK-regulated crown root development (Zhao et al. 2009, 2015b; Zhou et al. 2017). The loss of function or down-regulation of *OsWOX11* results in a significant reduction in the number and elongation of crown roots, whereas overexpressing this gene significantly promotes crown root growth and dramatically increases root biomass. Further data showed that OsWOX11 directly represses *OsRR2*, a type-A CK-responsive gene expressed in the crown root primordia, by binding to its promoter (Zhao et al. 2009). Moreover, OsERF3, an OsWOX11 interacting protein, positively regulates the expression of rice *RESPONSE REGULATOR 2* (*RR2*) during crown root initiation; the OsERF3-OsWOX11 interaction likely represses *OsRR2* expression during crown root elongation (Zhao et al. 2015b). OsWOX11 can also recruit the ADA2-GCN5 histone acetyltransferase to form a complex that commonly targets and regulates a set of root-specific genes involved in energy metabolism, cell wall biosynthesis, and the hormone response in the crown root meristem (Zhou et al. 2017). In addition, studies on another two genes, *CRL5* and *CK OXIDASE/DEHYDROGENASE 4* (*OsCKX4*), have provided further evidence that CK signalling plays an important role in crown root emergence and development (Kitomi et al. 2011; Gao et al. 2014). *CRL5* encodes an AP2/ERF transcription factor, expressed in the stem region where crown roots are initiated, and can be induced by exogenous auxin. The loss-of-function mutant *crl5* produced fewer crown roots and its initiation of crown root primordia was impaired. Further data show that CRL5 positively regulates the type-A RR, *OsRR1,* to repress CK signalling, indicating that *CRL5* integrates auxin and CK signalling to positively regulate crown root initiation in rice (Kitomi et al. 2011). *OsCKX4*, a CK oxidase/dehydrogenase (CKX) family gene, also plays a positive role in crown root formation; overexpressing *OsCKX4* was found to increase the number of crown roots but reduce the overall height of the plant. *OsCKX4* is a direct target of both the auxin response factor OsARF25 and the CK response regulators OsRR2 and OsRR3 (Gao et al. 2014). Consistant with this, overexpressing another RR gene, *OsRR6*, also suppressed root and vegetative development (Hirose et al. 2007). These data indicate that crown root development is co-ordinately regulated by auxin and CK signalling.

Increasingly, other plant hormones such as brassinosteroids (BRs) and SL have been implicated in crown root initiation and development. The *brd1* mutant possesses a dysfunctional BR biosynthesis gene, *OsBR6ox*, and produces fewer crown roots (Mori et al. 2002). The *dwarf* (*d*) mutants with impaired SL biosynthesis and signalling produce fewer adventitious roots than the WT (Arite et al. 2012). Application of GR24, a synthetic SL analogue, increases the number of adventitious roots in the SL-deficient mutant *d10*, but not in the SL-insensitive mutants *d3* and *d14*, indicating that adventitious root production is positively regulated by SL in rice (Sun et al. 2015).

Though many genes involved in rice crown root development have been identified (Fig. 1), our knowledge of this process is still fragmented, and its molecular mechanisms still require substantial elucidation.

### Genes Controlling LR Development

LRs are one of the most important root components, as they increase the root biomass, enabling the plant to absorb more water and nutrients as well as providing better anchorage in the soil. The molecular mechanisms regulating LR development are well studied in *Arabidopsis*, which revealed a major role for auxin signalling in this process (Benkova and Bielach 2010); however, LR development has not yet been well elucidated in rice, although significant progress has recently been made. Previously, two mutants, *lateral rootless 1 (lrt1*) and *lrt2*, were identified by their lack of LRs, and were found to be less sensitive to auxin (Chhun et al. 2003a; Wang et al. 2006; Faiyue et al. 2010). The *altered lateral root formation 1* (*alf1*) mutant also formed significantly shorter LRs and was less sensitive to exogenous auxin than the WT (Debi et al. 2003). Additionally, two other auxin-resistant recessive mutants, *auxin resistant mutant 1* (*arm1*) and *arm2*, failed to produce LRs (Chhun et al. 2003b). These findings suggest that auxin signalling indeed participates in LR formation in rice; however, the corresponding genes have not been reported, with the exception of *LRT2*. Previous results indicate that *OsCel9A*, a rice glycoside hydrolase family gene, plays an essential role in regulating auxin-induced LR primordia formation (Yoshida et al. 2006). Additionally, *NARROW LEAF 2* (*NAL2*) and *NAL3*, a pair of duplicated genes encoding WUSCHEL-RELATED HOMEOBOX 3A (OsWOX3A), also mediate LR development via the auxin signalling pathway. The *nal2 nal3* double mutant showed a severe reduction in LR number and an increase in root hair number and length, and the expression of the *OsPIN* genes was also significantly affected (Cho et al. 2013). Consistant with this, two IAA family genes, *OsIAA11* and *OsIAA13*, were reported to regulate LR formation, as LR development is inhibited in the gain-of-function mutants *Osiaa11* and *Osiaa13*; however, the other root components, including the crown roots and root hairs, are not affected (Kitomi et al. 2012; Zhu et al. 2012). A recent study further indicates that the inositol polyphosphate kinase OsIPK2 interacts with OsIAA11 to protect it from degradation and thereby inhibits lateral root formation (Chen et al. 2017). Another IAA family gene, *OsIAA23*, is specifically expressed in the quiescent centre cells of the root tip during the development of primary, lateral, and crown roots, and the gain-of-function mutant *Osiaa23* exhibited a pleiotropic phenotype, producing no crown root, no LRs, and no root cap (Ni et al. 2011).

Using a forward genetic approach, *OsCYCLOPHILIN 2* (*OsCYP2*) was cloned and functionally identified, and the loss-of-function mutant *Oscyp2* was found to exhibit defects in LR formation (Kang et al. 2013). Map-based cloning revealed that *lrt2* is allelic to *Oscyp2* (Zheng et al. 2013). OsCYP2 is a cyclophilin-type peptidyl-prolyl cis/trans isomerase that efficiently catalyses the cis/trans isomerization of OsIAA11 and directly regulates its stability; therefore, the *OsCYP2* mutation reduces the interaction between OsTIR1 and OsIAA11, causing the accumulation of OsIAA11 and inhibiting auxin signalling-mediated LR development (Jing et al. 2015). A recent study demonstrated that OsZFP, a C_2_HC-type zinc finger protein, can interact with OsCYP2 in the nucleus to regulate LR development (Cui et al. 2017). Furthermore, the inhibition of LR initiation was also reported in the auxin influx transporter mutant, *Osaux1* (Zhao et al. 2015a). These results indicate that auxin is a very important regulator of LR development in rice.

Other plant hormone signalling pathways have also been reported to influence LR growth and development; for example, *HEME OXYGENASE 1* (*HOX1*) is regulated by jasmonic acid (JA) to control the formation of LRs through the production of carbon monoxide (Chen et al. 2012; Hsu et al. 2012). Transgenic plants overexpressing *DROUGHT STRESS RESPONSE-1* (*OsDSR-1*), the *Arabidopsis* ortholog of which encodes a putative calcium-binding protein, produce much shorter LRs when grown in media containing ABA, suggesting that OsDSR-1 may act as a positive regulator during the ABA-mediated inhibition of LR development (Yin et al. 2011).

Moreover, many genes function in other physiological pathways involved in LR development in rice. *SHORT LATERL ROOT LENGTH 1* (*SLL1*), encoding a stearoyl-acyl carrier protein from the fatty acid desaturase family, affects overall fatty acid desaturation and also functions as a positive regulator of LR growth (Shelley et al. 2013). A pivotal factor in DNA replication, ORIGIN RECOGNITION COMPLEX SUBUNIT 3 (OsORC3), is also essential for LR development, as the *Osorc3* mutant exhibited a lateral rootless phenotype in a temperature-dependent manner (Chen et al. 2013).

### Similarities and Differences in the Molecular Mechanisms of Crown Root and Lateral Root Development in Rice

The developmental processes of crown roots and LRs are both tightly controlled by endogenous genetic programs that determine cell fate acquisition, cell division, and root primordia initiation, emergence, and elongation. Studies of rice mutants have led to the identification of many genes involved in LR and crown root development (Additional file 1: Table S1), some of which are involved in both processes while others are specifically involved one or the other. *OsIAA23*, *OsARM1*, *OsARM2*, and *CRL4*/*OsGNOM1* play essential roles in both LR and CR development (Chhun et al. 2003b; Kitomi et al. 2008; Liu et al. 2009; Ni et al. 2011), while *OsCYP2*/*OsLRT2*, *OsIAA11*, *OsIAA13*, and *OsLRT1* are required for LR initiation but not for the development of crown roots (Chhun et al. 2003a; Wang et al. 2006; Faiyue et al. 2010; Kitomi et al. 2012; Zhu et al. 2012; Kang et al. 2013). *OsCAND1* is required for crown root emergence but does not affect LR development (Wang et al. 2011). *OsCRL5* and *OsWOX11* are all required for the initiation of crown root development, but have no effect on LR development (Zhao et al. 2009; Kitomi et al. 2011). *ARL1*/*CRL1* mainly affects crown root development (Liu et al. 2005). Consistant with this, the loss-of-function mutant of the ARL1/CRL1 ortholog in maize, *rootless concerning crown and seminal root* (*rtcs*), exhibited defective crown root and embryonic seminal root initiation, but the development of the primary and lateral roots were not affected (Taramino et al. 2007). Notably, both the promoter regions of *RTCS* and its duplicated homologous gene *RTCS-LIKE* (*RTCL*) contain an auxin responsive element (ARE), suggesting that they are responsive to auxin just like *ARL1*/*CRL1* (Taramino et al. 2007). It suggests that ARL1/CRL1 and its orthologs have conserved functions in crown root initiation and development in monocot cereals. LBD16 and LBD29, the two most closely related homologs of ARL1/CRL1 in *Arabidopsis*, are direct targets of ARF7 and ARF19 and positively regulate LR formation (Okushima et al. 2007). This observation suggests that the LBD proteins might play different roles in the dicot *Arabidopsis* and the monocots rice and maize. Furthermore, recent studies have indicated that rice CRL1 could positively regulate 277 genes, including key genes in meristem patterning, cell proliferation, hormone homeostasis, and LR formation (Coudert et al. 2011, 2015), suggesting that the crown root and lateral root may share some regulatory pathway.

In rice, the Auxin–OsIAA11/OsIAA13 module functions as an important negative regulator of LR formation. A similar module also exists in *Arabidopsis*. A dominant negative mutant of *SLR/IAA14*, *slr1*, showed no lateral root initiation (Fukaki et al. 2005, 2010). IAA14 interacts with ARF7 and ARF19 and negatively regulates LR formation (Fukaki et al. 2005; Vernoux et al. 2011), indicating that auxin stimulates lateral root initiation through the SLR/IAA14–ARF7/ARF19 signalling module. Consistently, a second auxin-signalling module involving IAA12 and ARF5 was also shown to control lateral root initiation together with SLR–ARF7/ARF19 (De Smet et al. 2007). These data suggest a conserved function of Auxin–IAA–ARF in promoting LR formation in *Arabidopsis* and rice.

### Genes Controlling Root Hair Development

Root hairs are long tubular outgrowths that form on the surface of specialized epidermal cells. They are required for the uptake of nutrients and water, particularly in upland conditions. Root hair development can be divided into three phases: cell specification, initiation, and elongation (Cavell and Grierson 2000). The outgrowth of root hairs is strictly regulated by genetic and environmental factors. The first root-hairless mutant reported in rice was *rh2* (Suzuki et al. 2003). An exogenous application of NAA could induce very short root hairs in *rh2*, suggesting that the absence of root hairs in this mutant may be due to a shortage of endogenous auxin; however, the gene has not yet been identified. *OsWOX3A* was reported to control root hair formation through the regulation of auxin transport genes (Yoo et al. 2013), further suggesting that auxin is required for root hair elongation. Root hairs are initiated normally in the *Oscellulose synthase-like d1* (*Oscsld1*) mutant; however, this gene, which is expressed only in root hair cells, is required for their elongation (Kim et al. 2007; You et al. 2011). OsRHL1, a bHLH transcription factor expressed specifically in root hair cells, also regulates root hair elongation in rice; the loss-of-function mutants *Osroot hairless 1–1* (*Osrhl1–1*) and *Osrhl1–2* produced very short root hairs without affecting root length or the number of LRs and adventitious roots, suggesting that OsRHL1 functions specifically in root hair elongation (Ding et al. 2009). Furthermore, *OsFORMIN HOMOLOGY 1* (*OsFH1*) was also found to regulate rice root hair elongation; the loss-of-function mutant *osfh1* exhibited root hair defects when grown submerged in solution, but produced normal root hairs in contact with the air. This root hair phenotype could not be rescued by an external supply of hormones or carbohydrates (Huang et al. 2013a). It is well known that root hair growth requires extensive cell wall modification, and recent research revealed that *OsEXPA17*, encoding an expansin involved in cell wall remodelling, plays a crucial role in root hair elongation. *OsEXPA17* is exclusively expressed in root hair cells, and its null mutant forms short root hairs (Yu et al. 2011). Another rice gene, *OsSEC14-NODULIN DOMAIN-CONTAINING PROTEIN 1* (*OsSNDP1*), encoding a phosphatidylinositol transfer protein (PITP), promotes root hair elongation via phospholipid signalling and metabolism, suggesting that the mediation of these processes by PITP is required for root hair elongation in rice (Huang et al. 2013b). Additionally, transgenic rice overexpressing *STRESS/ABA-ACTIVATED PROTEIN KINASE 10* (*SAPK10*) produced longer root hairs, while plants overexpressing *OsABI-LIKE 2* (*OsABIL2*) had attenuated ABA signalling and shorter root hairs (Wang et al. 2017), suggesting that ABA is also responsible for root hair elongation.

Up to now, almost all of the genes reported to be involved in root hair development regulate their elongation; thus, the genes regulating root hair cell specification and initiation in rice are yet to be identified.

## Conclusion and Discussion

Faster and more extensive root growth is important for plant survival in complex soil conditions (de Dorlodot et al. 2007), as larger root systems typically enable plants to extract more water and nutrients from the soil (King et al. 2003). Root growth angle is also an important trait that affects rice RSA and the associated nutrient and water uptake. *DEEPER ROOTING 1* (*DRO1*) is believed to regulate RSA by regulating the growth angle of the crown roots to adapt to drought conditions; higher levels of *DRO1* expression result in deeper rooting that may maintain higher yields under drought conditions (Uga et al. 2013). *LARGE ROOT ANGLE 1* (*LRA1*), encoding OsPIN2, was also recently identified to regulate root growth angle in rice; the *lra1* mutant displays a shallow root system, which may benefit nutrient uptake (Wang et al. 2018). A more thorough understanding of the key genes involved in RSA and their regulation should enable breeders to breed cultivars with enhanced root systems using marker-assisted selection in the future.

Significant progress has been made in our understanding of the genetic control of root development in rice, particularly through the use of mutants with specific defects in root development and through the identification of QTLs using genetic linkage analyses. The identification of genes that regulate root traits will pave the way for more detailed genetic and molecular analyses of root system development in rice and other cereals. Though many genes that function in root development have been identified (Fig. 1; Additional file 1: Table S1), our knowledge about the molecular mechanisms of root elongation, crown root development, LR development, and root hair formation is still fragmented. The majority of studies have focussed mainly on identifying individual genes using mutants or QTL analyses, which limits our systemic understanding of the mechanisms of root development. With the rapid development of molecular biological approaches, the combination of functional genomics, transcriptomics, proteomics, and phenomics will rapidly expand our understanding of the molecular mechanisms controlling RSA.

Though many genes with key roles in RSA have been identified, little is known about the genetic improvement of the root system using these genes; can they be used in breeding? Cloning genes from QTL analyses is one feasible way to identify putative candidates for use in molecular breeding; however, it is difficult to identify QTLs with minor effects. *DRO1* and *PSTOL1* were both cloned from QTL analyses, and have been successfully used in breeding to improve root systems for drought tolerance and improve low-Pi tolerance for growth in less fertile soils (Gamuyao et al. 2012; Uga et al. 2013). Genome-wide association studies (GWAS) have been demonstrated to be a feasible and practical method to explore the alleles of existing varieties or genetic resources, which can then be applied in breeding. Recent studies using these approaches have identified several major QTLs containing promising candidate genes for root formation and development (Courtois et al. 2013; Biscarini et al. 2016; Bettembourg et al. 2017). However, both QTL analysis and GWAS have two main difficulties in cloning the genes associated with root development (Mai et al. 2014). First, it is difficult to do root phenotyping in real soil conditions, and the root phenotypes obtained from hydroponic and gel/agar systems do not really reflect their growth in soil. The second difficulty is the lack of precision in localizing QTLs using mapping populations or, to a much lesser extent, with association panels, because the root phenotype is more prone to vary in different growth conditions compared with the aboveground traits. Applying new approaches such as X-ray micro–computed tomography and magnetic resonance imaging to study the root system architecture in natural soils and in complex environments would enable more reliable measurement of root traits and the identification of related genes.

## Additional File

Additional file 1: **Table S1.** Identified genes that control root growth and development in rice. (DOC 122 kb)

## Abbreviations

ABA: Abscisic acid
ABIL2: ABA insensitive like 2
AFB2: Auxin signaling F-box 2
ALF: Altered lateral root formation
ARE: Auxin response element
ARF: Auxin response factor
ARL1: Adventitious rootless 1
ARM: Auxin resistant mutant
AUX/IAA: Auxin/Indole-3-Acetic acid
bHLH: Basic helix-loop-helix
BRs: Brassinosteroids
CAND1: Cullin-associated and neddylation-dissociated 1
CHR4: Chromatin remodelling 4
CK: Cytokinin
CKX4: CK oxidase/dehydrogenase 4
CRL: Crown rootless
CSLD1: Cellulose synthase-like D1
CYP2: Cyclophilin 2
DGL1: Dolichyl diphosphooligosaccharide-protein glycosyltransferase 1
DRO1: Deeper rooting 1
DSR-1: Drought stress response-1
EL5: Elicitor 5
ERF2: Ethylene responsive factor 2
EXPA: Expansion
FH1: Formin homology 1
GLU3: Membrane-bound endo- 1,4-β-glucanase
GNOM1: Membrane-associated guanine-nucleotide exchange factor of the ADP-ribosylation factor G protein (ARF-GEF)
HOX1: Heme oxygenase 1
JA: Jasmonic acid
LRA1: Large root angle 1
LRs: Lateral roots
LRT: Lateral rootless
MOGS: Mannosyl-oligosaccharide glucosidase
NAA: Naphthylacetic acid
NAL: Narrow leaf
ORC3: Origin recognition complex subunit 3
PIN: Pin-formed auxin efflux carrier proteins
PSTOL1: Phosphorus-starvation tolerance 1
QC: Quiescent center
QTL: Quantitative trait loci
RAM: Root apical meristem
RHL: Root hairless
ROS: Reactive oxygen species
RPK1: Receptor protein kinase 1
RR: Response regulator
RSA: Root system architecture
SAPK10: Stress/ABA-activated protein kinase 10
SL: Strigolactones
SLL1: Short later root length 1
SNDP1: Sec14-nodulin domain-containing protein
TIR1: Transport inhibitor response 1
TOP1: Topoisomerase 1
WOX: Wushel-related homeobox
WT: Wild type
