# Supplementary material for: Molecular Mechanisms of Root Development in Rice
Source: Rice (N Y). 2019 Jan 10;12:1. doi: 10.1186/s12284-018-0262-x (PMC6328431; doi:10.1186/s12284-018-0262-x)
Supplement: Supplementary file 1 — Table S1. Identified genes that control root growth and development in rice. (DOC 122 kb) [file 12284_2018_262_MOESM1_ESM.doc]

Table S1. Identified genes that control root growth and development in rice

|  | **Gene name** | **Gene ID** | **Biological function /process** | **Mutant**  **/Transgenic line** | **Root phenotype** | **Reference** |
| --- | --- | --- | --- | --- | --- | --- |
| *1* | *OsbHLH120* | Os09g0455300 | Salt stress | IRAT109 | Increased root length and thickness | Li et al. 2015a |
| *2* | *OsGNA1* | Os09g0488000 | De-novo UDPN-acetylglucosamine biosynthesis | *Osgna1* | Reduced length of PR and AR at high temperature | Jiang et al. 2005 |
| *3* | *Os**MOGS* | [Os01g0921200](http://rapdb.dna.affrc.go.jp/viewer/gbrowse_details/irgsp1?name=Os01g0921200;feature_id=478261) | N-glycosylation | *Osmogs* | Reduced length of PR, AR, LR; Fewer RHs | Wang et al. 2014a |
| *4* | *OsDGL1* | Os07g0209000 | N-glycosylation | *Osdgl1* | Reduced length of PR, AR, LR | Qin et al. 2013 |
| *5* | *OsCYT-INV1* | Os02g0550600 | Sugar signaling | *Oscyt-inv1* | Reduced length of PR, AR and LR | Jia et al. 2008 |
| *6* | *OsGLR3.1* | Os02g0117500 | Glutamate (Glu) receptors in Glu-activated ion channels | *Osglr3.1* | Reduced length of PR, AR and LR | Li et al. 2006 |
| *7* | *OsEXPA8* | Os01g0248900 | Cell wall loosening | *OsEXPA8-RNAi* | Reduced length of PR and AR;  Fewer LRs and no RHs | Wang et al. 2014b |
| *8* | *OsGatB* | Os11g0544800 | tRNA-dependent amidotransferase | *Osgatb* | Reduced length of PR and AR; Fewer LR | Qin et al. 2016 |
| *9* | *OsASL1* | [Os03g0305500](http://rapdb.dna.affrc.go.jp/viewer/gbrowse_details/irgsp1?name=Os03g0305500;feature_id=484622) | Arginine biosynthetic pathway | *Osasl1* | Retarded PR and AR growth | Xia et al. 2014 |
| *10* | *OsEL5* | [Os02g0559800](http://rapdb.dna.affrc.go.jp/viewer/gbrowse_details/irgsp1?name=Os03g0305500;feature_id=484622) | Activates nitrogen signaling | *mEL5* | Reduced length of PR and AR | Koiwai et al. 2007 |
| *11* | *OsSPR1* | Os01g0898300 | Ion homeostasis | *Osspr1* | Reduced length of PR,AR and LR | Jia et al. 2011 |
| *12* | *OsMYB1* | Os01g0128000 | Pi signaling and GA biosynthesis | *myb1* | Reduced length of LR;  Length of PR is not responsive to low Pi | Gu et al. 2017 |
| *13* | *OsARF12* | Os04g0671900 | Auxin signaling | *Osarf12T*, *Osarf12*,  *Osarf12 ⁄ 25* | Reduced length of PR and AR | Qi et al. 2012 |
| *14* | *miR393* | OsJ_00328 | Auxin signaling | *miR393a-OX*, *miR393b-OX* | Increased length of PR and AR | Bian et al. 2012 |
| *15* | *OsTOP1* | Os08g0154600 | Auxin signaling | *OsTOP1-RNAi* | Reduced length of PR and AR | Shafiq et al. 2017 |
| *16* | *OsEIL1* | [Os03g0324200](http://rapdb.dna.affrc.go.jp/viewer/gbrowse_details/irgsp1?name=Os03g0324200;feature_id=484748) | Ethylene signaling | *OsEIL1-OX* | Reduced length of PR and AR | Mao et al. 2006 |
| *17* | *OsERF2* | [Os06g0181700](http://rapdb.dna.affrc.go.jp/viewer/gbrowse_details/irgsp1?name=Os06g0181700;feature_id=494519) | Ethylene signaling | *nsf2857* (gain of function mutant),  *AmiOsERF2* | Reduced length of PR in *sf2857*;  Increased length of PR in *AmiOsERF2* | Xiao et al. 2016 |
| *18* | *OsAIM1* | [Os02g0274100](http://rapdb.dna.affrc.go.jp/viewer/gbrowse_details/irgsp1?name=Os06g0181700;feature_id=494519) | Salicylic acid biosynthesis | *aim1* | Reduced length of PR and AR | Xu et al. 2017 |
| *19* | *SHB* | [Os05g0389000](http://rapdb.dna.affrc.go.jp/viewer/gbrowse_details/irgsp1?name=Os05g0389000;feature_id=492364) | GA Biosynthesis | *shb* | Reduced length of PR | [Li et al. 2015](#_ENREF_10)b |
| *20* | *ARL1/CRL1* | Os03g0149100 | Auxin signaling | *arl1*, *crl1* | No AR or CR | Liu et al. 2005;  Inukai et al. 2005 |
| *21* | *OsYUCCA1* | Os01g0645400 | Auxin signaling | *OsYUCCA1-OX* | Increased number of CR | Yamamoto et al. 2007 |
| *22* | *CRL4/*  *OsGNOM1* | Os03g0666100 | Auxin signaling | *gnom1-1*, *gnom1-2*, *gnom1-3*, *gnom1-4, Oscrl4* | Reduced length of PR;  Reduced number of CR and LR | Liu et al. 2009  Kitomi et al. 2008 |
| *23* | *OsPIN1* | Os02g0743400 | Auxin signaling | *OsPIN1-RNAi*,  *OsPIN1-OX* | Increased length of PR and  increased number of LR in *OsPIN1-OX*;  Reduced number of CR in *OsPIN1-RNAi* | Xu et al. 2005 |
| *24* | *OsIAA23* | Os06g0597000 | Auxin signaling | *Osiaa23* | Increased length of PR; Reduced number of AR and no LR;  Defective gravitropic response | Ni et al. 2011 |
| *25* | *OsNAL1* | Os04g0615000 | Auxin signaling | *nal1*, *nal5* | Reduced length of PR and AR;  Reduced number of AR | Cho et al. 2014 |
| *26* | *OsCAND1* | Os02g0167700 | Auxin signaling | *Oscand1* | Reduced length of PR and AR;  No AR | Wang et al. 2011 |
| *27* | *OsCHR4/*  *CRL6* | Os07g0497100 | Auxin signaling;  Chromatin remodeling | *crl6*, *Oschr4* | Reduced number and length of CR | Wang et al. 2016; Zhao et al. 2012 |
| *28* | *OsRPK1* | [Os05g0486100](http://rapdb.dna.affrc.go.jp/viewer/gbrowse_details/irgsp1?name=Os05g0486100;feature_id=492982) | Auxin signaling | *OsRPK1-OX* | Reduced length of PR;  Reduced number of AR;  Reduced density of LR | Zou et al. 2014 |
| *29* | *OsWOX11* | Os07g0684900 | Auxin or cytokinin signaling | *Oswox11* | Reduced length of PR and CR;  Reduced number of CR | Zhao et al. 2009 |
| *30* | *OsERF3* | Os01g0797600 | Auxin or cytokinin signaling | *OsERF3-OE*,  *OsERF3-ami* | Reduced length of PR and reduced number of CR in *ami*;  Increased length of PR and increased number of CR in *OE* | Zhao et al. 2015b |
| *31* | *OsADA2* | [Os03g0750800](http://rapdb.dna.affrc.go.jp/viewer/gbrowse_details/irgsp1?name=Os03g0750800) | Auxin or cytokinin signaling | *OsADA2–RNAi* | Reduced length of PR and CR;  Reduced number of CR | Zhou et al. 2017 |
| *32* | *OsGCN5* | [Os10g0415900](http://rapdb.dna.affrc.go.jp/viewer/gbrowse_details/irgsp1?name=Os10g0415900) | Auxin or cytokinin signaling | *OsGCN5–RNAi* | Reduced length of PR and CR; Reduced number of CR | Zhou et al. 2017 |
| *33* | *OsCRL5* | Os07g0124700 | Auxin or cytokinin signaling | *crl5* | Reduced length of PR and CR;  Reduced number of CR; Defective gravitropic response | Kitomi et al. 2011 |
| *34* | *OsCKX4* | Os01g0940000 | Cytokinin signaling | *ren1-D* | Increased length of PR and CR;  Increased numbers of CR | Gao et al. 2014 |
| *35* | *OsRR6* | Os04g0673300 | Cytokinin signaling | *OsRR6-OX* | Reduced length of PR and CR | [Hirose et al. 2007](#_ENREF_5) |
| *36* | *BR6ox* | Os03g0602300 | Brassinosteroid signaling | *brd1* | Reduced length of PR, CR,LR;  Reduced number of CR and LR | Mori et al. 2002 |
| *37* | *OsWOX3A* | Os12g0101600 | Auxin signaling | *nal2/3* | Reduced member of LR;  Reduced length of LR | Cho et al. 2013;  Yoo et al.2013 |
| *38* | *OsIAA11* | Os03g0633500 | Auxin signaling | *Osiaa11* | Increased length of PR and AR;  Reduced number of LR | Zhu et al. 2012 |
| *41* | *OsIAA13* | Os03g0742900 | Auxin signaling | *Osiaa13* | Increased length of PR;  Reduced density of LR and RH | Kitomi et al. 2012 |
| *42* | *OsCYP2/* *OsLRT2* | Os02g0121300 | Auxin signaling | *cyp2-1,*  *lrt2* | Increased length of PR and CR;  Reduced number and length of LR | Kang et al. 2013  Zheng et al. 2013 |
| *43* | *OsAUX1* | Os01g0856500 | Auxin signaling | *Osaux1-1,*  *Osaux1-2,*  *Osaux1-3* | Increased length of PR and AR;  Reduced length and number of LR | Yu et al. 2015;  Zhao et al. 2015a |
| *44* | *OsSLL1* | Os04g0379900 | Fatty acid desaturation | *Ossll1* | Reduced length and number of LR | Shelley et al. 2013 |
| *45* | *OsORC3* | Os10g0402200 | DNA replication | *Osorc3* | Reduced length of PR and LR at high temperature;  Reduced number of LR and RH at high temperature | Chen et al. 2013 |
| *46* | *OsCSLD1* | Os10g0578200 | Auxin signaling | *OsCSLD1::Ds* | Reduced length of PR;  Reduced length and number of RH | Kim et al. 2007 |
| *47* | *OsRHL1* | Os06g0184000 | Transcriptional regulating | *Osrhl1-1*, *Osrhl1-2* | No RH | Ding et al. 2009 |
| *48* | *OsEXPA17* | Os06g0108600 | Cell-wall modification | *Osexpa17* | Reduced length of RH | Yu et al. 2011 |
| *49* | *OsFH1* | Os01g0169800 | Actin-dependent transport regulating | *Osfh1-1, Osfh1-2,*  *Osfh1-3* | Reduced length of RH | Huang et al. 2013a |
| *50* | *OsSNDP1* | Os10g0122600 | PITP-mediated phospholipid signaling and metabolism | *Ossndp1* | Reduced length of RH | Huang et al. 2013b |
| *38* | *OsSAPK10* | [Os03g0610900](http://rapdb.dna.affrc.go.jp/viewer/gbrowse_details/irgsp1?name=Os03g0610900;feature_id=485711) | ABA signaling | *OsSAPK10-OX*, | Increased length of RH | Wang et al. 2017 |
| *39* | *OsABIL2* | [Os05g0592800](http://rapdb.dna.affrc.go.jp/viewer/gbrowse_details/irgsp1?name=Os05g0592800;feature_id=493841) | ABA signaling | *OsABIL2-OX* | Reduced length of RH | Wang et al. 2017 |
| *51* | *OsPSTOL1* | AB45844.1 | Pi starvation signaling | *OsPSTOL1-OX*  *Pub1* | Increased root length and root surface area | Gamuyao et al. 2012 |
| *52* | *OsDRO1* | Os09g0439800 | Auxin signaling | *Dro1-NIL* | Reduced root growth angle | Uga et al. 2013 |
| *53* | *OsLRA1* | Os06g0660200 | Auxin signaling | *lra1* | Increased root growth angle | Wang et al. 2018 |

Note: PR, primary root; AR, adventitious root; CR, crown root; LR, lateral root; RH, root hair; OX, overexpression line; RNAi , RNA interferring line; Ami，artificial microRNA-mediated silenced line; NIL, near isogenic line.
